# Anchor questions to improve patient-reported outcome measure interpretability in patients undergoing knee or hip arthroplasty - a mixed-methods content validity, construct validity, and reliability study

**DOI:** 10.1007/s11136-025-03987-y

**Published:** 2025-05-16

**Authors:** Lasse K. Harris, Trine S. Larsen, Berend Terluin, Henrik H. Lauridsen, Anders Troelsen, Lina H. Ingelsrud

**Affiliations:** 1 Department of Orthopaedic Surgery, Copenhagen University Hospital Hvidovre, Copenhagen, Denmark; 2 Department of Clinical Medicine, Faculty of Health and Medical Sciences, University of Copenhagen, Copenhagen, Denmark; 3 Department of Clinical Research, Copenhagen University Hospital, Hvidovre, Copenhagen, Denmark; 4 Department of People and Technology, Roskilde University, Roskilde, Denmark; 5 Department of General Practice, Amsterdam UMC Location, Vrije Universiteit Amsterdam, Amsterdam, The Netherlands; 6 Amsterdam Public Health Research Institute, Amsterdam, The Netherlands; 7 Department of Sports and Clinical Biomechanics, University of Southern Denmark, Odense, Denmark

**Keywords:** Content validity, Construct validity, Reliability, Anchor question, Knee arthroplasty, Hip arthroplasty

## Abstract

**Purpose:**

To explore content validity, construct validity, and reliability of anchor questions used to determine minimal important change (MIC), patient acceptable symptom state (PASS) and treatment failure (TF) in patients undergoing knee or hip arthroplasty.

**Methods:**

A mixed-methods study from one public hospital. Evaluation of content validity involved applying thematic analysis to data from think-aloud interviews. To ascertain construct validity and reliability, we focused on patients who underwent surgery between 2016 and 2022 and had responded to preoperative and either 3-, 12- or 24-month postoperative questionnaires. Confirmatory factor analysis (CFA) was employed to assess present state bias (PSB), model fit, and reliability of the anchor questions.

**Results:**

We conducted 18 interviews with patients aged 52 to 84 (10 female). Based on seven emerging themes from the content validity analysis, MIC and PASS anchor questions were considered relevant and comprehensible, while the TF anchor question had several problems. Data from 1197 to 2207 patients, with 3-, 12-, or 24-month postoperative responses, were used to evaluate construct validity. The median age was 69-70 years (56-59% female). PSB for MIC was between 54 and 73%, and reliability for the anchor questions was between 0.52 and 0.80 for all time points. The CFA models varied between good and poor fit.

**Conclusion:**

The MIC and PASS anchor questions demonstrated a high degree of content validity, while it was questionable for TF. Construct validity was considered good to poor for PASS, but patients may consider their present state more than their preoperative state when responding to the MIC. Reliability was considered acceptable in both MIC and PASS.

**Supplementary Information:**

The online version contains supplementary material available at 10.1007/s11136-025-03987-y.

## Introduction

Knee and hip arthroplasty are cost-effective alternatives to relieve patients with end-stage knee/hip osteoarthritis of their pain and functional limitations [1, 2]. Patient-reported outcome measures (PROMs) are commonly used to evaluate patients’ own experienced outcomes after surgery [3]. While PROMs provide valuable insights, their interpretation can be challenging. For example, determining the clinical significance of a 10-point change in a PROM score can be difficult [4]. Interpretation thresholds have been introduced to address this issue. The minimal important change (MIC) is a threshold for meaningful within-person change. When this threshold is exceeded, average patients perceive themselves as importantly changed [5, 6]. On the other hand, the patient acceptable symptom state (PASS) and treatment failure (TF), are thresholds related to the patient’s current well-being [7, 8]. The PASS represents the PROM score at follow-up above which patients consider their symptoms satisfactory. The TF represents the PROM score at follow-up below which patients consider their treatment to have failed. The MIC, PASS, and TF thresholds are derived using anchor-based approaches in which the PROM score is anchored to an external measure. The external measure is often a question, asking patients to evaluate their perceived degree and importance of improvement or their perceived current health state. Each threshold requires its own specific anchor question [9].

Several studies have aimed to improve anchor questions, but debates persist over the best way to formulate these questions, with various studies exploring different approaches to their design [10–14]. A previous initiative, aimed to achieve consensus on specific wording, emphasised the importance of examining the impact of different anchors [15]. According to contemporary guidance, the anchor question should align with the domain/construct measured by the PROM. Additionally, the anchor should be able to classify patients who have achieved minimal but important improvement while remaining understandable and relevant to the patients [16, 17].

Despite progression in refining anchor questions, gaps remain in evaluating their content validity, construct validity, and reliability. Assessing whether anchor questions are relevant and comprehensible for patients informs their degree of content validity, which necessitates obtaining input directly from patients [18]. Furthermore, anchor questions that evaluate change over time may be influenced more by patient’s follow-up state than by their actual change experienced, an effect known as present state bias (PSB) [19–21]. Evaluating anchor questions’ PSB necessitates conducting a confirmatory factor analysis (CFA) that models the anchor questions’ responses with the PROM under study. These analyses subsequently provide insights into the anchor questions’ degree of construct validity [22]. Another consideration is that single questions have lower reliability than multi-item questionnaires [23]. This raises concerns about whether anchor questions are reliable and to what extent [24]. Therefore, we explored content validity, construct validity, and reliability of anchor questions used to determine MIC, PASS, and TF in patients undergoing knee and hip arthroplasty.

## Methods

### Design

This mixed-methods content and construct validation study used individual patient interviews and prospective registry data from a local arthroplasty database at one public hospital in Denmark. In the quantitative part, evaluation methods were used that were recently suggested by one of the authors of this paper [21, 25]. Reporting of the registry-based data was guided by the Strengthening the Reporting of Observational Studies in Epidemiology (STROBE) checklist [26]. Reporting of the qualitative study was guided by the consolidated criteria for reporting qualitative research (COREQ) 32-item checklist [27].

### Participants

Patient interviews to inform content validity of the anchor questions were conducted between November 2022 and January 2023. Patients were eligible if they had undergone primary knee/hip arthroplasty at the hospital and had completed a preoperative questionnaire. They were recruited for this study within 4, 8, or 12 weeks before their scheduled 3-, 12-, or 24-month follow-up questionnaire, respectively. We sampled purposively and recruited patients with a variation across the preoperative (Oxford Knee/Hip Score [OKS/OHS] grouped from 0 to 16 points, 17–32 points, and 33–48 points), follow-up time point (i.e., 3-, 12-, or 24-month postoperatively), age (i.e., younger to older), gender (i.e., male or female) and arthroplasty received (i.e., knee or hip).

To inform construct validity and reliability of the anchor questions, data from a single-centre arthroplasty database that included patients undergoing knee/hip arthroplasty between February 2016 and December 2022 was used. Patients completed an electronic questionnaire during their preoperative visit to the hospital, and electronic follow-up questionnaires were emailed at 3-, 12-, and 24-month postoperatively. Paper versions were posted if the patient preferred. Two reminder emails were sent two weeks apart, and one reminder was sent to those preferring postal versions. The inclusion criterion was patients undergoing primary surgery for knee/hip arthroplasty. If patients had bilateral arthroplasty, we selected the first for inclusion.

### Instruments

The preoperative questionnaire included the widely used OKS or OHS [28], which are 12-item (5-point Likert scale) questionnaires evaluating pain level and function, with summarised scores ranging from 0 (worst) to 48 (best). They have been proposed as reliable, responsive, and content and construct valid PROMs utilised in major arthroplasty registries worldwide [29]. Additionally, the unidimensionality of the OKS and OHS scales has been confirmed in validation studies using item response theory or CFA [30, 31]. Danish translations of the questionnaires are available, but cross-cultural adaption and validation have only been tested for the OHS in Danish settings [32]. The postoperative questionnaire included the OKS or OHS [28] and the MIC, PASS, and TF anchor questions [6–8] (Table 1). Various anchor questions have been used in the literature, but no consensus exists on the best anchor [4, 9].

**Table 1:** Anchor items used to determine minimal important change, patient acceptable symptom State, and treatment failure criteria

Anchor Questions	Anchor Response and Classification of Response Options
Minimal Important Change • How are your knee/hip problems now compared to prior to your operation?	Importantly improved 1: Better, an important improvement 2: Somewhat better, but enough to be an important improvement Unchanged 3: Very small improvement, not enough to be an important improvement 4: About the same 5: Very small deterioration, not enough to be an important deterioration Importantly deteriorated 6: Somewhat worse, but enough to be an important deterioration 7: Worse, an important deterioration
Patient Acceptable Symptom State	
• Taking into account all the activities you have during your daily life, your level of pain, and also your functional impairment, do you consider that your current state is satisfactory?	1: Yes 2: No
Treatment Failure	
• If you answered “no” to the previous question, would you consider your current state as being so unsatisfactory that you think the treatment has failed?	1: Yes 2: No

### Content validity

#### Data collection

The individual interviews used the Three-Step Test Interview (TSTI) observation-based method [33], designed to pre-test questionnaires and identify issues with survey questions or response processes. The method introduces the ‘think aloud’ technique, where participants complete questionnaires, while verbalising their thoughts, producing observational data about their immediate reactions. This step ensures the data is respondent-driven without interviewer influence. In step two, verbal probing clarifies thought processes and potential misunderstandings, making the data interviewer-driven. Step three involves a semi-structured debriefing interview, capturing respondents’ experiences, overall impressions, and remaining issues. This structured combination and interview methods enables insights into participants’ thinking and reflections [33].

#### Interview guide

We developed a semi-structured interview guide to investigate patients’ perception and understanding of the standardised questionnaire they received at 3-, 12-, and 24-month after knee/hip arthroplasty at the hospital. The standardised questionnaire comprised 15 questions, the 12-item OKS or OHS and the three anchor questions (Table 1) [6–8, 28]. The guide consisted of two sections. The first section introduced and prepared patients to use the ‘think aloud’ technique. The first 12 questions in the standardised questionnaire were used to practise the ‘think aloud’ technique. In the second section, patients used the technique on the three anchor questions and were afterwards debriefed about their opinions and experience with open-ended questions. LKH, TSL, BT, HHL, and LHI developed a draft version of the interview guide. We adjusted the guide twice after pilot-test interviews with four people with various knee/hip problems related to osteoarthritis and two people with knee osteoarthritis involved as patient representatives.

#### Interviews

Physiotherapist and PhD-student, LKH, conducted the pilot tests and the individual interviews. LKH had formal introductory MSc-level coursework, including education and patient interview training. Additionally, LKH received feedback and guidance from TSL, who was highly experienced in qualitative methodology with a PhD in social health science.

Patients were recruited via telephone calls. We aimed to conduct the interviews in the patients’ homes because it would be closest to the ‘real life’ experience of responding to the questionnaire. One patient preferred another setting, so we used a soundproof meeting room at the hospital, located separate from the department where they had been admitted. We sought to collect data until data saturation, meaning no new key findings occurred with additional interviews [34]. In total, 13 were “not interested” and 18 completed individual interviews lasting between 30 and 60 min. During interviews, LKH made field notes about non-verbal expressions and/or contextual details to add to the transcripts. The interviews were recorded and transcribed verbatim before analysis. Further details about the research team, reflexibility, and study design are presented (Online Resource 1).

#### Analysis

We conducted a thematic analysis using a six-phase analytical process, moving from familiarising with the data, generating codes through a detailed engagement with the data, searching for themes, reviewing themes, ultimately defining themes, and writing up the overall analysis [35].

The initial coding process was data-driven (inductively). The next step was guided by existing theory, as reflected in the interview questions (deductively). Two analytical questions were used to guide the coding process: (1) the relevance and comprehensibility of the anchor questions and (2) the relevance and comprehensibility of the response options. During the inductive coding process, we generated codes across the entire material (employing a transversal analysis approach) and subsequently applied a more deductively coding process in which ‘relevancy’ and ‘comprehensibility’ were concepts to help explain the data in this process [35]. The combined inductive and deductive coding process was underlined by concrete statements. Codes were clustered into meaningful patterns from where we identified central themes. These themes were then organised, referring to each anchor question or the transversal analyses.

Analyses were performed by LKH and guided by TSL and LHI, who supervised the analysis using several meetings to discuss the coding process and construction of analytical themes, findings, and interpretation. The analyses were performed iteratively, repeating a process of reflection and discussion until a consensus on themes and interpretation was reached. Microsoft Word was used to manage data and we did not engage in participant feedback for our findings.

### Construct validity and reliability

We applied novel methods to evaluate the construct validity and reliability of the anchor items. Whether the construct of the anchor question matches the construct of the OHS and OKS was evaluated qualitatively, informed by interviews with the patients. The statistical construct evaluation of the single-item anchor question is evaluated in relation to the constructs of the OHS and OKS. Importantly, an essential component of the MIC anchor question is to evaluate change over time. Using a novel methodology, we assessed the degree of PSB using CFA as one measure to inform the construct validity of the MIC anchor [21]. Additionally, reliability was also assessed using a novel CFA-based method rather than the traditional test-retest design [25]. In a classical test theory perspective, reliability is the ratio between the true score variance and the observed score variance. The CFA approach used has been shown to correctly estimate the reliability of single-item anchor questions [25].

#### Analysis

CFA was used to estimate PSB and reliability in separate models for knee and hip arthroplasty cohorts at each postoperative time point. Reliability of the three anchor questions was estimated separately for the three anchor questions and PSB solely for the MIC anchor question since it evaluates change over time [25]. Ordinal item CFA using weighted least square mean and variance-adjusted estimators was applied in all models [36].

The degree of PSB was assessed by fitting a correlated two-factor longitudinal CFA model with the 12 OKS or OHS items at baseline loading on factor 1, the same items at follow-up loading on factor 2, and the MIC anchor item loading on both factors [21]. To capture the latent change and follow-up trait (means and variances) relative to the baseline trait, the model is identified by standardising the baseline factor and constraining the OKS/OHS items’ factor loadings and thresholds [21]. The PSB statistic represents the proportion of the follow-up trait that is included in the “perceived change” instead of the true change [21]. A PSB value higher than 50% implies that the follow-up state has a more decisive influence on the MIC anchor response than the actual change experienced [21]. As an acceptable fit of the model to the data is important to capture the latent change and follow-up state accurately, model fit was evaluated and improved by model modifications if necessary. Model fit was assessed by the comparative fit index (CFI), Tucker-Lewis index (TLI), and root mean square error of approximation (RMSEA) values [37, 38]. CFI or TLI values above 0.95 demonstrate good fit [38], and RMSEA values below 0.01, 0.05, and 0.08 indicate excellent, good, and mediocre fit, respectively [37]. In case of unacceptable model fit, cross-sectional item correlations were included if indicated by modification indices and considered clinically relevant. Additionally, if necessary, we explored measurement invariance (or response shift) of the OKS/OHS items over time by freeing item factor loadings and thresholds. A backward approach was used, first by releasing factor loading constraints for one item at a time and second by releasing threshold constraints for one item at a time. Improvement of the model fit through freeing an item parameter, as evidenced by a Bonferroni adjusted p-value < 0.002 and a CFI change > 0.001 was taken as indicating non-invariance. Using this strategy, we tested models for the three postoperative time points separately, and any item that was flagged for non-invariance at any time point was treated as non-invariant in the final model. We additionally performed a sensitivity analysis, treating only items flagged for non-invariance at all time points as non-invariant.

We used a similar correlated two-factor longitudinal CFA model to assess the MIC anchor question reliability [25]. The model was identified by standardising both factors, while no constraints were imposed on the factor loadings and thresholds. Reliability was thus estimated as the R^2^ of the anchor question and represents the proportion of the variance of the anchor explained by the latent change that it is assumed to measure [25]. This CFA model was adapted for the PASS and TF anchor questions, as a one-factor model using only the postoperative time point [25]. Model fit was evaluated using the fit indices mentioned above [37, 38]. Since there are no definite criteria for good reliability, we used R^2^ ≥ 0.50 to indicate acceptable reliability for the anchor questions.

We also calculated biserial correlations to measure the relationship between dichotomised MIC anchors and the OKS or OHS change scores and between PASS and TF anchors and the postoperative OKS or OHS values. The appropriate correlation between the anchor and PROM used has been debated to be ≥ 0.30–0.50 [9, 39, 40].

Furthermore, we obtained 95% confidence intervals (CIs) for PSB using nonparametric (emperical) bootstrapping (*n* = 1.000).

Finally, depending on their distribution, baseline characteristics are presented as the mean and standard deviation (SD) or median and interquartile range (IQR) for continuous variables and as frequencies and percentages for categorical variables. We used the software program R, version 4.1.2, for all statistical analyses [41].

## Results

### Content validity

In total, 18 out of 31 (58%) invited patients were interviewed. Patients’ ages ranged from 51 to 84 years, and 56% were female (Table 2). Typically, patients who declined the interview invitation were younger and more often male than those interviewed (Online Resource 2).

**Table 2:** Patient preoperative characteristics for exploring content validity. Numbers are median (min - max) unless otherwise stated

Factor		Knee surgeries	Hip surgeries
		*n* = 9	*n* = 9
Age		72 (55–84)	68 (51–77)
Sex % (n) female		56% (5)	56% (5)
BMI		31 (21–31)	26 (23–37)
ASA classification % (n)			
	1	-	-
	2	78% (7)	89% (8)
	3	22% (2)	11% (1)
	4	-	-
OKS/OHS		25 (9–35)	20 (4–39)
EQ-5D-3 L		0.72 (0.23–0.78)	0.66 (-0.14-0.78)
EQ VAS		71 (30–90)	70 (13–89)
Days until follow-up*		42 (3–56)	49 (0–65)

* Number of days from being interviewed until patients were scheduled to receive their follow-up questionnaire

BMI; Body Mass Index, ASA; American Society of Anaesthesiologists physical status classification system, 1; normal health, 2; mild systemic disease, 3; severe systemic disease, 4; severe systemic disease that is a constant threat to life, OKS; Oxford Knee Score, OHS; Oxford Hip Score, VAS; Visual Analog Scale

Generally, patients perceived the MIC and PASS anchor questions as relevant and comprehensive. In contrast, there was variation in how the TF anchor question was perceived, and some patients questioned its relevance at 3 months after surgery. Seven themes emerged from the thematic analysis that provided further insight into the content validity: (1) ‘MIC: pain and function’, (2) ‘PASS: a complex evaluation’, (3) ‘TF: ambiguous wording’, (4) ‘temporal aspect’, (5) ‘order and interrelation’, (6) ‘emotions’, and (7) ‘current health state’. A brief overview of the themes is presented below, and quotes are presented to support the analysis (Table 3). A detailed description of the analysis is also available (Online Resource 3).

**Table 3:** Patient themes, supporting quotes, and interpretation for the content validity analysis of the three anchor questions

Themes	Quotes	Interpretation
1) MIC: pain and function	**Anchor question relevancy and comprehensibility**:	
	*“It has provided me with so much quality of life. From being unable to walk very far and taking many painkillers*,* to now being able to climb stairs and ladders*,* get up and down from the floor*,* uh… Yes*,* to not have pain when standing up. In other words*,* my whole life has changed.” (P18)*	Demonstrates that patients interpret the MIC question through their pain level and functional limitations, which influence their quality of life.
	*“I think this is a relevant question… which I think you have to ask (…) how my symptoms have been remedied.” (P5)*	Patients directly affirm the relevance of the MIC question, emphasising that symptom change is central to evaluating treatment outcomes.
	**Response options relevancy and comprehensibility**:	
	*“Initially*,* I was concerned until I reread them (referring to the response options). The reason was that they were not all important to me. But when I properly read them through*,* I can see that there is a big difference*,* whether it is an important improvement or not an important improvement.” (P5)*	Highlight that patients distinguish between levels of improvement and appreciate the nuance provided by the response options, even if they are initially overwhelmed by the number of choices.
2) PASS: a complex evaluation	**Anchor question relevancy and comprehensibility**:	
	*“It does not state that it is only related to the knee… in my head*,* it can be perceived that way because of all the other disabilities I have.” (P1)*	Illustrates how patients may interpret the question as referring to their overall health, not just the joint, revealing possible ambiguity in scope.
	*“I respond based on what I do… spend time taking care of my horse*,* which takes up a lot of my day… Those who invented the question*,* what do they want to know? Is it about daily activities*,* grooming*,* or being able to clean the house and cook dinner?” (P12)*	Demonstrates how patients associate the question with pain during daily activities while also highlighting the complexity and broad interpretation of terms like “activities”.
	**Response options relevancy**:	
	*“It can hardly be anything other than yes and no… it will be too difficult to add the response option ‘perhaps’… Because I think it will cause people to get more confused.” (P9)*	Highlight that the binary response format appears straightforward and that including more response options would likely cause confusion.
3) TF: ambiguous wording	**Anchor question relevancy and comprehensibility**:	
	*“How I was received at the hospital department… Prelude to the surgery. The conversation with the doctor. The day of the surgery… The follow-up afterwards… the progress with the physiotherapist.” (P12)*	Suggests that patients may interpret TF differently, not just the surgery itself but the entire treatment continuum, which can result in varied interpretations.
	*“And then I should not pick the response option ‘yes’. This was why I reread it again… It is a reverse question… therefore*,* you could make a mistake here too.” (P16)*	Highlights that the negative phrasing of the TF question is confusing, causing some patients to reread or misinterpret the intent.
	**Response options relevancy**:	
	*“If you wait 4*,* 5*,* 6 months after the surgery. I think you could get a correct response… This thing… I have gone through (talks about the surgery)*,* was it worth the effort… I cannot say anything about that yet. And that is why I say ‘no’ to the question.” (P4)*	Reflects that patients 3 months after surgery may feel it is too soon to assess whether treatment has failed.
4) Temporal aspect	*“It depends a lot on the timing of when you receive this questionnaire… there is a big difference between the condition now and in*,* say*,* 12 months.” (P2)*	Confirms that the postoperative timing affects patient responses as their condition and perceptions evolve.
	*“But I expect it to be temporarily (laughs). It should very much be so… I have been told it can take up to a year with a knee like this before I am back again.” (P4)*	Patients expect ongoing recovery and, therefore, find it difficult to judge failure of treatment as soon as three months after surgery.
5) Order and interrelation	*“And that is*,* in fact*,* similar to what I have responded in the first questions (refer to the 12 Oxford Hip Score questions).” (P5)*	Illustrates that patients compare and connect questions throughout the survey, which may influence their interpretation.
6) Emotions	*“Maybe if you could elaborate it a bit*,* like right now where we sit and talk together… that you would be able to write down a bit of text.” (P12)*	Reflects a desire to express nuanced experiences that do not fit within closed-ended formats, hinting at emotional frustration.
	*“This is why I have been so much against all these questionnaires because I do not feel they can describe specifically what I mean. You cannot always do that with yes and no questions*,* and how is it from 1 to 10 response options.” (P14)*	Indicates emotional discomfort with a limited response format, which may impact engagement and response accuracy.
7) Current health state	*“It can be difficult to distinguish between the right and left hip because now I have severe pain across my lower back. It is the left hip*,* in particular*,* causing the problem*,* and it is the most recent surgery I have undergone.” (P14)*	Identifies that overlapping health issues may obscure patients’ ability to isolate responses to a specific joint.
	*“But I am also very old*,* 86. Something always comes around when you are this old (referring to illnesses and general health problems).” (P1)*	Demonstrates how a patient’s current health state and age may influence their interpretation and response to questions.

MIC; minimal important change, PASS; patient acceptable symptom state, TF; treatment failure

#### MIC: pain and function

Patients found the MIC anchor question relevant and easy to comprehend. They described ‘knee/hip problems’ as comprehensive constructs involving their pain level, which was closely related to their functional limitations, which in turn influenced their quality of life. They responded based on their current situation, recollecting how they felt before surgery and assessed whether their perceived outcome corresponded to their expectations. Furthermore, many found the MIC response options understandable and intuitively sound. Nevertheless, some patients expressed that careful consideration was necessary to distinguish between the response options, as they covered both the magnitude and the importance of the change. These individuals were able to select a response option that they found suitable, thoroughly considering their current state in comparison to their preoperative condition and whether they considered their improvement lived up to their expectations.

#### PASS: a complex evaluation

Patients considered the PASS anchor question important and relevant but acknowledged its complexity. They considered their daily activities, pain level, and functional impairment when evaluating their current condition. Despite its complexity, their responses primarily focused on the interplay between pain and daily activities, making it challenging to delineate these aspects. Additionally, they raised the issue that “knee/hip” was not specified, which they believed might lead some individuals to respond based on their overall health condition rather than their surgery-specific outcomes. Furthermore, many patients suggested adding more response options, but they did so by considering other people’s needs rather than their own. None had difficulties choosing between the current response options.

#### TF: ambiguous wording

Patients confirmed that exploring why treatment fails is relevant but interpreted the TF anchor question differently. ‘Treatment’ varied from meaning the surgical intervention to other parts of the whole treatment continuum, while ‘failure’ was difficult to evaluate without specifying what it refers to. Additionally, some misinterpreted the question because it was negatively worded, with “no” being the favourable answer. Finally, we identified a *temporal aspect* for some patients undergoing knee/hip arthroplasty within the past three months. These patients had difficulties responding to the question and did not consider it relevant because they considered it too early to judge whether they considered the treatment to have failed, as they expected a recovery time of up to a year.

#### Temporal aspect

The identified temporal aspect of the TF anchor question, questioned its relevancy at 3 months postoperatively, and led to investigate whether there was a similar time-dependency of the MIC and PASS anchor questions. The temporal feature was not confirmed for the MIC and PASS anchor questions, which seem to be read and perceived constantly over time.

#### Order and interrelation

Patients fostered their perceptions of questions and ways of responding by comparing them to each other; some may be negatively affected by varying complexity. Furthermore, some patients preferred familiarity and consistency across questions.

#### Emotions

Patients’ frustrations with the questionnaire were related to a desire to elaborate and could potentially undermine the sincerity of their responses. Some strongly preferred including free-text fields to address this issue effectively.

#### Current health state

Patients’ health may affect how they perceive and respond to questions regarding their recent knee/hip arthroplasty. Someone with additional health problems, undergoing other treatment, subsequent surgery, or surgery in another joint may have difficulty explicitly distinguishing the impact of the current arthroplasty intervention.

### Construct validity and reliability

We obtained 3-, 12-, or 24-month complete data from 857 to 1499 (66–73%) patients undergoing knee or hip arthroplasty (Online Resource 4). Patients were median 69–70 years old, and 56–59% were female (Table 4). Generally, preoperative PROM scores reflected better health states for respondents to postoperative questionnaires compared to non-respondents across the knee and hip arthroplasty cohorts (Online Resource 5).

**Table 4:** Patient preoperative characteristics for exploring construct validity and reliability. Numbers are median (2.5–97,5% quantile range) unless otherwise stated

Factor		Knee surgeries			Hip surgeries		
		3-month *n* = 1040	12-month *n* = 1499	24-month *n* = 1337	3-month *n* = 857	12-month *n* = 886	24-month *n* = 955
Age		70 (62–75)	69 (61–75)	69 (62–74)	70 (62–76)	70 (62–76)	70 (61–75)
Sex % (n) female		59% (614)	58% (875)	59% (782)	57% (490)	56% (497)	58% (550)
BMI		29 (26–33)	29 (26–33) ^a^	29 (26–33) ^a^	27 (24–31)	27 (24–31)	27 (24–30)
ASA classification % (n)							
	1	7.2% (75)	9.2% (138)	10.3% (138)	15.1% (129)	15.1% (134)	15.3% (146)
	2	73.4% (763)	72.9% (1093)	72.6% (970)	63.2% (542)	65.8% (583)	67.4% (644)
	3	19.1% (199)	17.6% (264)	16.8% (225)	21.6% (185)	19.1% (169)	17.1% (163)
	4	0.3% (3)	0.3% (4)	0.3% (4)	0.1% (1)	-	0.2% (2)
OKS/OHS		22 (17–27)	22 (18–27)	22 (18–27)	21 (16–26)	21 (16–27)	22 (17–27)
EQ-5D-3 L		0.66 (0.59–0.72) ^a^	0.70 (0.59–0.72) ^b^	0.66 (0.59–0.72) ^b^	0.66 (0.56–0.72)	0.66 (0.56–0.72)	0.66 (0.56–0.72)
EQ VAS		66 (50–80) ^b^	70 (50–80) ^c^	70 (50–80) ^d^	60 (45–78) ^b^	60 (49–80) ^b^	61 (50–80) ^b^

^a^ Missing data *n* = 1, ^b^ Missing data *n* = 2, ^c^ Missing data *n* = 6, ^d^ Missing data *n* = 7

BMI; Body Mass Index, ASA; American Society of Anaesthesiologists physical status classification system, 1; normal health, 2; mild systemic disease, 3; severe systemic disease, 4; severe systemic disease that is a constant threat to life, OKS; Oxford Knee Score, OHS; Oxford Hip Score, VAS; Visual Analog Scale

The PSB for the MIC anchor question for knee and hip arthroplasties, respectively, was 0.56 (CI, 0.41 to 0.76) and 0.54 (CI, 0.34 to 0.72) at 3 months, 0.73 (CI, 0.59 to 0.88) and 0.61 (CI, 0.36 to 0.84) at 12 months, and 0.60 (CI, 0.45 to 0.74) and 0.55 (CI, 0.30 to 0.80) at 24 months postoperatively. Overall, CFA model fit was good to mediocre, with CFI and TLI ranging from 0.954 to 0.980 and RMSEA ranging from 0.048 to 0.061. Reliability increased across the three time points, ranging between 0.64 and 0.80 and 0.62 and 0.77 for knee and hip arthroplasties, respectively, with good to mediocre model fit (Table 5). Sensitivity analysis to explore measurement invariance flagged some OKS and OHS items but did not alter the PSB estimates (Online Resource 6). For the PASS and TF anchor questions, CFA model fit ranged from good to poor, with CFI and TLI ranging from 0.974 to 0.987 and RMSEA ranging from 0.072 to 0.090. Reliability ranged between 0.52 and 0.75, and the biserial correlations ranged between 0.31 and 0.70 across time points and arthroplasties. Both increased with the length of follow-up (Table 5).

**Table 5:** Present state bias (PSB), model fit indices, reliability, and biserial correlation for minimal important change (MIC), patient acceptable symptom state (PASS), and treatment failure (TF) anchor questions and the Oxford knee and hip score (Change) scores at 3, 12, and 24 months after knee or hip arthroplasty

Factor	Knee arthroplasty			Hip arthroplasty		
	3 months *n* = 1039	12 months *n* = 1490	24 months *n* = 1333	3 months *n* = 857	12 months *n* = 886	24 months *n* = 952
**MIC**						
PSB ^a^	0.56 (0.41–0.76) ^c^	0.73 (0.59–0.88) ^c^	0.60 (0.45–0.74) ^c^	0.54 (0.34–0.72) ^d^	0.61 (0.36–0.84) ^d^	0.55 (0.30–0.80) ^d^
CFI	0.967	0.979	0.980	0.960	0.969	0.977
TLI	0.962	0.976	0.977	0.954	0.964	0.973
RMSEA	0.054	0.048	0.049	0.061	0.059	0.053
Reliability	0.64	0.71	0.80	0.62	0.76	0.77
CFI	0.967	0.980	0.980	0.949	0.961	0.969
TLI	0.962	0.977	0.977	0.942	0.956	0.965
RMSEA	0.054	0.048	0.049	0.069	0.066	0.061
Cor change ^b^	0.46 (0.41–0.51)	0.49 (0.45–0.53)	0.55 (0.51–0.59)	0.38 (0.32–0.44)	0.39 (0.33–0.44)	0.44 (0.39–0.49)
**PASS**						
Reliability	0.56	0.75	0.75	0.52	0.62	0.68
CFI	0.979	0.987	0.986	0.980	0.983	0.983
TLI	0.974	0.984	0.982	0.975	0.979	0.979
RMSEA	0.079	0.074	0.078	0.077	0.083	0.090
Cor postop ^b^	0.55 (0.51–0.59)	0.70 (0.67–0.73)	0.69 (0.66–0.72)	0.53 (0.48–0.58)	0.63 (0.59–0.67)	0.68 (0.65–0.71)
**TF**						
Reliability	0.52	0.62	0.71	0.54	0.56	0.64
CFI	0.981	0.988	0.985	0.982	0.982	0.983
TLI	0.976	0.994	0.981	0.977	0.978	0.978
RMSEA	0.075	0.072	0.079	0.074	0.083	0.090
Cor postop ^b^	0.35 (0.30–0.40)	0.51 (0.47–0.55)	0.56 (0.52–0.60)	0.31 (0.25–0.37)	0.44(0.39–0.49)	0.52 (0.47–0.56)

^a^ Values in parentheses are 95% confidence interval, calculated using 1000-replication bootstrapping and reported as 0.025-to-0.975 quantiles

^b^ Values in parentheses are 95% confidence interval

^c^ Factor loading constrained items 2, 5, 8, 10, 12, and threshold constrained items 8

^d^ Factor loading constrained items 4, 6, 8, 11, and threshold constrained items 8

CFI; comparative fit index, TLI; Tucker-Lewis index, RMSEA; root mean square error of approximation, Cor; correlation, postop; postoperative

## Discussion

In this mixed-methods study, we used semi-structured individual observation-based think-aloud interviews and registry data from patients undergoing knee/hip arthroplasty at one public hospital in Denmark to explore content validity, construct validity, and reliability of anchor questions used to determine MIC, PASS, and TF. Using a thematic analysis, we identified that patients generally perceived the MIC and PASS anchor questions as relevant and comprehensible. However, patients may subconsciously prioritise their current health state when responding to the MIC anchor question rather than judging their true change in relation to the surgery. Similar indications of PSB were found using CFA analyses. Overall, CFA models suggested good to mediocre fit for all anchor questions. Additionally, reliability estimates were considered acceptable for all anchor questions. Lastly, the TF anchor question was interpreted in multiple ways and considered irrelevant to some after surgery.

### MIC

The validity of transition items, such as the MIC anchor question, is primarily determined by their alignment with the intended construct (e.g., pain and function), although their interpretation may vary across different populations and treatments. After elective orthopaedic surgery, patients considered the question relevant and comprehensible, wanting to compare their current and preoperative state. Our findings that the MIC question adequately captured the constructs measured by the OKS and OHS (knee or hip pain and function) also confirms the comprehensiveness of the anchor. Patients’ considerations when responding to the MIC anchor question reflected these domains, indicating that the anchor was sufficiently broad to encompass relevant aspects of joint function without being overly general. Our findings inform the discussion about transition items, which are often criticised for being subject to recall bias beyond four weeks [9, 24, 40]. However, four weeks after knee/hip arthroplasty, patients are most often still affected by their surgically induced trauma. Therefore, soliciting patients to evaluate their change in such a short timeframe after surgery is not appropriate. Previous qualitative evaluation of transition questions have questioned their validity because patients valued their current health status more than their true change [19, 42]. Furthermore, such confounding due to comorbidity was similarly found in patients with chronic respiratory or heart diseases, highlighting that problems related to other conditions may override the condition under study when patients are prompted to evaluate their change. Similarly, in our study, a few patients indicated that other health problems may have impacted on their judged change. Additionally, Taminiau-Bloem and colleagues emphasised that even though patients who received radiation therapy for cancer seemed to make a comparison of their current to their former health state, the applied timeframe was often inconsistent with what was intended. We did not replicate those findings from the interviews, probably because the surgical intervention is such a prominent event, making it natural for patients to recollect and evaluate their perceived change from before to after surgery. However, the construct validity analysis did indicate PSB. Unlike prior studies [21, 40], our findings suggest that PSB did not increase over time, indicating that patients’ recollections of their preoperative state remained stable for up to two years postoperatively. Furthermore, the measurement invariance testing indicated that PSB estimates were not critically dependent on the perfect fit of the model. These findings suggest a need to reassess the causes and mechanisms of PSB, which the context of significant interventions like life-changing surgeries may influence. Finally, our findings suggest that patients measure their perceived outcome against their expectations, which aligns with previous findings [43]. Clinicians play an essential part in addressing patients’ expectations before surgery, as patients often have diverse expectation levels, which influence their perception of their treatment outcome.

### PASS

Judging whether one’s health status is satisfactory is a complex evaluation. There is a paucity in the literature on the content validity of PASS anchor questions. Previously, a special-interest group in rheumatic disorders discussed the PASS concept, concluding that more work was needed before reaching a consensus on the wording of the anchor question [15]. Our study contributes to this discussion by presenting the perspective of patients undergoing knee/hip arthroplasty. Patients found the question important and relevant but also complex. They pointed at one crucial modification: the specific condition under study needs to be specified (i.e., asking about “knee/hip state”) to avoid responses based on their overall health rather than their current knee/hip condition. A revised question would read: *“Taking into account all the activities you have during your daily life*,* your level of pain*,* and also your functional impairment*,* do you consider that your current knee/hip state is satisfactory?”*. The importance of specifying a transition question’s construct was likewise demonstrated in patients with rheumatoid arthritis [10]. Additionally, some patients considered the simple yes/no response options to provide a simplified evaluation of the complex ways in which pain and functional disability influence a patient’s daily life, as it cannot capture the varying degrees of impact or the interplay between these factors that patients may experience. Despite the complexity involved, the yes/no response format is desirable for estimating PASS values, for which it is essential to have a binary judgement of whether a symptom state is acceptable or not. Our interviews revealed that patients considered their pain in relation to their functional disability, confirming that the PASS anchor question construct matches the OKS and OHS construct. A close match between constructs has been pointed out as essential for anchor questions [17, 40]. The comprehensiveness of the PASS anchor is further supported by its alignment with key domains measured by the OKS and OHS, while providing a simple and interpretable threshold. However, we acknowledge that single-item anchors inherently involve trade-offs in capturing complex constructs, as they do not fully account for the nuances of patient experiences.

### TF

Content validity was questionable for the TF anchor question for several reasons. Although patients found it relevant to explore why treatment sometimes ‘fails’, it is difficult to define and thereby fully understand what is meant by ‘treatment failure’. Previous definitions of ‘failure’ in knee/hip surgery have been the need for revision surgery [41] or antibiotic treatment for infection after surgery [44]. Asking the patients directly was an attempt to measure the patient’s own perception of treatment failure [7]. However, we found that the domain ‘treatment failure’ is too ambiguously defined, leading to extensive variation in interpretation and incomparable responses. Therefore, we recommend focusing on PASS or patient-perceived treatment success rather than treatment failure. Already established TF values from published studies should be interpreted with caution.

### Strengths and limitations

This study has strengths. We used a mixed-methods approach on patients from the same population to facilitate a more comprehensive understanding of the anchor questions used to determine MIC, PASS, and TF. Additionally, the think-aloud method aimed to ensure the fidelity of interview data by initially allowing participants to respond without interviewer input, minimising interference. Subsequently, the interviewer probed to explore the participants’ reasoning and clarify their responses, ensuring a balance between neutrality and in-depth understanding [33]. Furthermore, LKH had limited experience in qualitative interviews and analysis. However, we thoroughly developed the interview guide, conducted supervised pilot interviews, and received continuous feedback on interviews and discussions with an experienced qualitative researcher during the analysis phase. Additionally, the many observations with prospectively collected data may minimise selection bias within the knee/hip arthroplasty population. However, our quantitative findings may have limited generalizability, as data were collected at one public hospital where 66–73% of patients undergoing knee/hip arthroplasty provided complete data. Importantly, the hospital serves a wide range of rural and urban areas, and the age and sex composition align with the nationwide population, which improves generalizability [45, 46]. Additionally, we acknowledge that we did not assess reliability using a traditional test-retest design. Such a study would help to understand the consistency of responses to the anchor questions over time. Instead, we used a CFA-based approach, which—while increasingly applied in recent methodological work—remains less established in clinical research [25]. The advantage of this model-based estimation of reliability is that it estimates the error associated with a single measurement, which is a requirement when calculating MIC values [47]. Lastly, reliability estimations for hip arthroplasty at 3 months for MIC and 12 and 24 months for PASS and TF were based on non-optimal model fit, despite our effort to improve the model fit as much as possible. However, in those cases where suboptimal models could be improved to acceptable models, we observed that the impact of suboptimal model fit on the estimates of MIC and PASS values was minimal.

## Conclusions

This study is the first to investigate the content validity of transition items in patients undergoing elective orthopaedic surgery an intervention that is markedly different from the treatment provided in previous studies on anchor questions. The MIC anchor question demonstrated a high degree of content validity and acceptable reliability, but patients’ responses may be more contingent on their current health state rather than reflecting their true change in health state related to the intervention explored. The PASS anchor question had a high degree of content validity, good to poor construct validity, and acceptable reliability, while the TF anchor question revealed questionable content validity. We recommend that registers and future studies carefully consider which anchor questions to use and how they are tailored to their respective patient populations. In our registry, the PASS anchor question was revised to specifically concern patients’ knee/hip condition, not their overall health and the TF anchor question was removed.

## Electronic supplementary material

Below is the link to the electronic supplementary material.

Supplementary Material 1

Supplementary Material 2

Supplementary Material 3

Supplementary Material 4

Supplementary Material 5

Supplementary Material 6

Supplementary Material 7

Supplementary Material 8
