# Supplementary material for: Anchor questions to improve patient-reported outcome measure interpretability in patients undergoing knee or hip arthroplasty - a mixed-methods content validity, construct validity, and reliability study
Source: Qual Life Res. 2025 May 16;34(8):2279–91. doi: 10.1007/s11136-025-03987-y (PMC12274218; doi:10.1007/s11136-025-03987-y)
Supplement: Supplementary file 3 — Supplementary Material 3 [file 11136_2025_3987_MOESM3_ESM.docx]

**Online Resource 1**

**Article title**Anchor questions to improve patient-reported outcome measure interpretability in patients undergoing knee or hip arthroplasty – A mixed-methods content validity, construct validity, and reliability study

**Journal name**Quality of Life Research

**Author names**
Lasse K. Harris^1,2^, Trine S. Larsen^1,3,4^, Berend Terluin^5,6^, Henrik H. Lauridsen^7^, Anders Troelsen^1,2^,
Lina H. Ingelsrud^1^

**Affiliations**
^1^ Department of Orthopaedic Surgery, Copenhagen University Hospital Hvidovre, Copenhagen, Denmark
^2^ Department of Clinical Medicine, Faculty of Health and Medical Sciences, University of Copenhagen, Denmark
^3^ Department of Clinical Research, Copenhagen University Hospital, Hvidovre, Copenhagen, Denmark
^4^ Department of People and Technology, Roskilde University, Roskilde, Denmark
^5^ Department of General Practice, Amsterdam UMC Location, Vrije Universiteit Amsterdam, the Netherlands
^6^ Amsterdam Public Health Research Institute, Amsterdam, the Netherlands
^7^ Department of Sports and Clinical Biomechanics, University of Southern Denmark, Odense, Denmark

**Corresponding author**Lasse K. Harris, E-mail: [lasse.kindler.harris@regionh.dk](mailto:lasse.kindler.harris@regionh.dk)

**COREQ 32-item checklist additional information**
LKH had no direct relationship with the participants before the commencement of the study. From previous employment as a physiotherapist, he has experience rehabilitating patients undergoing arthroplasty. This information was not shared with the participants. However, they were informed that LKH and the research team were very interested in understanding better how questionnaires are being interpreted and what patients think about while responding to specific questions after undergoing knee or hip surgery. Furthermore, participants were told that participation would and could not affect their previous or potential upcoming treatment in any way.

The assumption behind the study was that some questions have multiple ways of being interpreted by patients, which may lead researchers and clinicians to draw questionable conclusions. This information was not shared with the participants. During each interview, none other than the patient and LKH were present. Participants were informed that the interview would take no longer than 60 minutes. During the study, none of the patients dropped out. Interviews were not repeated, and patients were not presented with transcripts.
