# Supplementary material for: Anchor questions to improve patient-reported outcome measure interpretability in patients undergoing knee or hip arthroplasty - a mixed-methods content validity, construct validity, and reliability study
Source: Qual Life Res. 2025 May 16;34(8):2279–91. doi: 10.1007/s11136-025-03987-y (PMC12274218; doi:10.1007/s11136-025-03987-y)
Supplement: Supplementary file 4 — Supplementary Material 4 [file 11136_2025_3987_MOESM4_ESM.docx]

**Online Resource 2**

**Article title**Anchor questions to improve patient-reported outcome measure interpretability in patients undergoing knee or hip arthroplasty – A mixed-methods content validity, construct validity, and reliability study

**Journal name**Quality of Life Research

**Author names**
Lasse K. Harris^1,2^, Trine S. Larsen^1,3,4^, Berend Terluin^5,6^, Henrik H. Lauridsen^7^, Anders Troelsen^1,2^,
Lina H. Ingelsrud^1^

**Affiliations**
^1^ Department of Orthopaedic Surgery, Copenhagen University Hospital Hvidovre, Copenhagen, Denmark
^2^ Department of Clinical Medicine, Faculty of Health and Medical Sciences, University of Copenhagen, Denmark
^3^ Department of Clinical Research, Copenhagen University Hospital, Hvidovre, Copenhagen, Denmark
^4^ Department of People and Technology, Roskilde University, Roskilde, Denmark
^5^ Department of General Practice, Amsterdam UMC Location, Vrije Universiteit Amsterdam, the Netherlands
^6^ Amsterdam Public Health Research Institute, Amsterdam, the Netherlands
^7^ Department of Sports and Clinical Biomechanics, University of Southern Denmark, Odense, Denmark

**Corresponding author**Lasse K. Harris, E-mail: [lasse.kindler.harris@regionh.dk](mailto:lasse.kindler.harris@regionh.dk)

| **Supplementary table.** Preoperative characteristics of patients who declined invitation to explore content validity. Numbers are median (min - max) unless otherwise stated. | | | |
| --- | --- | --- | --- |
| **Factor** | | **Knee surgeries** | **Hip surgeries** |
|  |  | **n = 5** | **n = 8** |
| Age | | 66 (53-76) | 61 (52-76) |
| Sex % (n) female | | 40% (2) | 13% (1) |
| BMI | | 27 (26-37) | 27 (23-30) |
| ASA classification % (n) | |  |  |
|  | 1 | - | 13% (1) |
|  | 2 | 100% (5) | 63% (5) |
|  | 3 | - | 24% (2) |
|  | 4 | - | - |
| OKS/OHS | | 17 (9-28) | 23 (17-36) |
| EQ-5D-3L | | 0.63 (0.26-0.78) | 0.72 (0.56-0.99) ^a^ |
| EQ VAS | | 30 (11-74) | 75 (39-93) ^a^ |
| ^a^ Missing data n = 1. BMI; Body Mass Index, ASA; American Society of Anaesthesiologists physical status classification system, 1; normal health, 2; mild systemic disease, 3; severe systemic disease, 4; severe systemic disease that is a constant threat to life, OKS; Oxford Knee Score, OHS; Oxford Hip Score, VAS; Visual Analog Scale. | | | |
