# Supplementary material for: Anchor questions to improve patient-reported outcome measure interpretability in patients undergoing knee or hip arthroplasty - a mixed-methods content validity, construct validity, and reliability study
Source: Qual Life Res. 2025 May 16;34(8):2279–91. doi: 10.1007/s11136-025-03987-y (PMC12274218; doi:10.1007/s11136-025-03987-y)
Supplement: Supplementary file 5 — Supplementary Material 5 [file 11136_2025_3987_MOESM5_ESM.docx]

**Online Resource 3**

**Article title**Anchor questions to improve patient-reported outcome measure interpretability in patients undergoing knee or hip arthroplasty – A mixed-methods content validity, construct validity, and reliability study

**Journal name**Quality of Life Research

**Author names**
Lasse K. Harris^1,2^, Trine S. Larsen^1,3,4^, Berend Terluin^5,6^, Henrik H. Lauridsen^7^, Anders Troelsen^1,2^,
Lina H. Ingelsrud^1^

**Affiliations**
^1^ Department of Orthopaedic Surgery, Copenhagen University Hospital Hvidovre, Copenhagen, Denmark
^2^ Department of Clinical Medicine, Faculty of Health and Medical Sciences, University of Copenhagen, Denmark
^3^ Department of Clinical Research, Copenhagen University Hospital, Hvidovre, Copenhagen, Denmark
^4^ Department of People and Technology, Roskilde University, Roskilde, Denmark
^5^ Department of General Practice, Amsterdam UMC Location, Vrije Universiteit Amsterdam, the Netherlands
^6^ Amsterdam Public Health Research Institute, Amsterdam, the Netherlands
^7^ Department of Sports and Clinical Biomechanics, University of Southern Denmark, Odense, Denmark

**Corresponding author**Lasse K. Harris, E-mail: [lasse.kindler.harris@regionh.dk](mailto:lasse.kindler.harris@regionh.dk)

**Content validity analysis of three anchor questions using thematisation**

In this study, the content validity evaluation of the three anchor questions, minimal important change (MIC), patient acceptable symptom state (PASS), and treatment failure (TF), was informed by interviewing 18 patients who underwent knee or hip arthroplasty within the past two years at a Danish hospital. Interviews were transcribed and subsequently coded using an iterative process. Codes were generated both deductively and inductively. The deductive codes focused on patients’ perceptions of each anchor question's content, relevancy, and comprehensibility, resulting in three themes. The first theme describes that patients relate to the MIC anchor question by focusing on their level of pain and functional limitations. The second theme describes that patients consider the PASS anchor question as complex. The third theme describes that patients point at ambiguous wording of the TF anchor question, and their responses reflect that they perceive the words ‘treatment’ and ‘failure’ differently. The inductive analysis was made across the anchor questions, resulting in four emerging themes describing that different aspects affect how patients respond to the questionnaire. The first theme describes the temporal aspect when patients respond to these questions postoperatively. The second theme describes the significance of interrelation between questions and the order in which they are asked. The third theme describes that patients may act due to emotions that arise while reading the questions. The fourth and final theme describes that patients’ current health state affects how they perceive and respond to questionnaires in general.

| **Online Resource Table 1.** Deductive and inductive themes. | |
| --- | --- |
| **Deductive themes** | **Inductive themes** |
| MIC: Pain and function | Temporal aspect |
| PASS: A complex evaluation | Order and interrelation |
| TF: Ambiguous wording | Emotions |
|  | Current health state |

**MIC - Pain and function**The following section describes how patients read and understood the MIC anchor question and whether they found it relevant and meaningful. First, patients relate to pain and function when asked to compare their knee/hip from before surgery to their current state. Second, they emphasise and relate to the words ‘knee/hip problems’. Finally, they understand and relate to the response options.

MIC anchor question relevancy and comprehensibility
Patients broadly explained that the MIC anchor question, “How are your knee/hip problems now compared to prior to your operation?” asked them to compare their current knee or hip condition with their condition before surgery. P8 explained, *“You actually want to compare how I feel… before compared to after the surgery”*. The first thing most patients considered was the level of pain they felt before the surgery compared to today. P5 said, *“Most of my considerations are that I was tormented by pain before, and now I am not”*. In addition to the level of pain, the question was frequently associated with patients’ thoughts about their functional limitations. In general, patients talked directly and indirectly about their functional limitations, mostly in close relation to their level of pain. First, P6 said, *“I have been able to do many things, but some were too painful, and then it just kept me from doing them”*, and then P15 said, *“Previously, when I was in pain, I could not walk 100 meters. I could not even cross the road to go grocery shopping… and today I am happily doing it”*. Furthermore, some patients considered that their pain and functional improvements impacted their quality of life. P18 said, *“It has provided me with so much quality of life. From being unable to walk very far and taking many painkillers, to now being able to climb stairs and ladders, get up and down from the floor, uh… Yes, to not have pain when standing up. In other words, my whole life has changed”*.

Generally, patients found the MIC anchor question to be broad and relevant. For example, P5 talked about why the question is relevant, *“I think this is a relevant question… which I think you have to ask (…) how my symptoms have been remedied”*. In addition, word choice can be essential for the comprehensibility of the question. Some patients emphasised that interpreting the words ‘knee/hip problems’ is key to understanding the question. P4 explained that patients might interpret this concept broadly: "*It says knee problems. This can be anything related to function… up and down the stairs, jumping on a bike and things like that (…) It is a bit comprehensive… unless you are willing to get all sorts of weird interpretations of it”*. Additionally, P18 talked about that the concept already presumes that you have knee problems, which no longer need to be the case for everyone, *“The way the question is being asked, it already assumes that I have knee problems. It could be that I did not have any”* and suggested rephrasing the question by removing the word ‘problems’, *“Perhaps you could rephrase it… How do you perceive your knee today compared to before surgery?”*.

In summary, patients seemed to consider the MIC anchor question relevant and understandable. ‘Knee/hip problems’ were described as comprehensive constructs that involved their level of pain, which was closely related to their functional limitations, which in turn influenced their quality of life.

MIC response options relevancy and comprehensibility
Similarly, patients were asked to consider the MIC response options, “1. Better, an important improvement, 2. Somewhat better, but enough to be an important improvement, 3. Very small improvement, not enough to be an important improvement, 4. Unchanged, 5. Very small deterioration, not enough to be an important deterioration, 6. Somewhat worse, but enough to be an important deterioration, 7. Worse, an important deterioration”. Generally, patients explained that the response options were understandable and intuitively sound, which P10 talked about, *“Starts with improvement first. And unchanged afterwards. And then deterioration last. I actually think it is very well distributed. It cannot be any other way”*. Subsequently, there were mixed opinions and attitudes towards the content of the response options, including the number of options available. A large proportion of patients felt that the seven options were fine, as they contributed to a more nuanced understanding of the patient's perception of change, which P16 talked about, *“It is distributed into seven… That is all very well. Other questions that only need to be responded to with either yes or no are incomplete. In most cases it is not nuanced enough”*. Some patients thought that the difference between the response options was too narrow. P13 described it as something that may lead to doubtful responses, *“It is more so when you are about to respond… Then, it is a bit difficult to find the right response option… It does not completely hit the spot because some things cross into the next option. And then you get slightly hesitant about which one to pick”*. Additionally, some patients considered response options three and five the most challenging because of the transition between positive and negative wording. P17 said, *“Very small improvement, not enough to be an important improvement. In my world, it is duplicated… It is one of the difficult ones, the third option … I must respond positively to negatively (…) I think it is the same problem that exists for response options three and five … It is difficult because it is positive to negative”*. Meanwhile, some patients requested a response option to feeling completely relieved of their symptoms because they no longer notice their artificial joint, which P6 talked about, *“I almost feel like I am cured… There should be one option that says, I have been cured… all the other options are no longer relevant”.* And some patients thought that fewer options could make the response process more straightforward, which P14 talked about, *“I might not think as much if there were only three (referring to the number of response options)”*.

Some patients also suggested specific changes to how the MIC response options could be phrased. For example, P15 talked about how the repetitive wording may cause unnecessary confusion when choosing a response option, *“I think the third option is a bit tricky… that it says improvement two times in the same line, it might confuse me a bit… And again deterioration, an important deterioration, to me it is repetitions”*. In addition, P3 mentioned that the length of some of the response options could be readjusted to accommodate any uncertainties, *“It is a bit convoluted in a way… these last three words… They could have been omitted (…) you may get a bit uncertain about what exactly is meant by them”*. Additionally, P18 mentioned that the possibilities might seem trivial to read: “*The response options are kind of trivial when you read them… they are very boring to read… I was already bored halfway through”*. This statement could suggest a risk of patients becoming unfocused when dealing with many options that are not so different from each other. However, some patients also talked about the intention behind the wording of the response options. P5 explained that there is a big difference between being importantly improved or not, *“Initially, I was concerned until I reread them (referring to the response options). The reason was that they were not all important to me. But when I properly read them through, I can see that there is a big difference, whether it is an important improvement or not an important improvement”.* Similarly, P2 talked about that ‘important improvement’ are loaded words leading to individual interpretations, *“But what does an important improvement imply? You should try to avoid using loaded words… it leads to individual interpretations that you cannot compare”*. These statements confirm that some patients consider whether their change in symptoms is important or not to them, and how they judge ‘importance’ is individual.

In summary, the structure and intended meaning of the MIC response options are understandable and intuitively sound to patients. However, the transition between positive and negative wording may be challenging, and when the options are perceived to be close to each other, it can be difficult for some patients to decide which one to choose. Nevertheless, all the informants found an option suitable for their situation, although it took more thought process for some. Most were overall satisfied with the current options, but some preferred having fewer options with fewer words for simplicity.

**PASS – A complex evaluation**
The following section describes how patients read and understood the PASS anchor question and whether they found it relevant and meaningful. First, patients had different thoughts and opinions about how to respond to the questions about their current state. Second, some made an overall assessment of their condition rather than exclusively related to their new artificial knee/hip. Finally, they expressed that although it can be complex to consider daily life activities, pain level, and functional impairment in combination, choosing a response option was easy.

PASS anchor question relevancy and comprehensibility
Generally, patients had many different thoughts and opinions about the perception of the PASS anchor question, “Taking into account all the activities you have during your daily life, your level of pain, and also your functional impairment, do you consider that your current state is satisfactory?”. A widespread reaction from the patients was to consider their most important everyday activities. P9 said, *“I perceive this as to how I am doing in my everyday life… Concerning what I can do… For example, exercise, clean, read… And then how good or bad I handle it”*. Additionally, many patients considered their functional situation before surgery and compared it to their current situation. P10 explained that some things had become more challenging after the surgery: “*I feel like many of the things I usually do are more challenging today…. I am not able to do exactly the same things I once could”*. Although many patients focused on their physical condition, some compared the level of pain they felt before and after surgery. For some, it was difficult to separate pain and function because one presupposed the other. P17 said, *“I do not have pain in my knee anymore. And I think my functional impairment is related to pain because pain has hampered my function”*. In addition, some patients thought the question could be related to more than just their knee or hip surgery. P1 talked about how other competing disorders may have influenced the perception of the question, *“It does not state that it is only related to the knee… in my head, it can be perceived that way because of all the other disabilities I have”*. Therefore, some patients may perceive the questions as an overall assessment of their condition rather than exclusively their new artificial knee/hip joint, leading to unintentional responses.

Generally, patients found that the PASS anchor question was broad and relevant, which was expressed by P3, *“It is one of the good questions… I mean, it is all the things you wanted to do before that you were unable to”* and then by P13, *“It is a very broad question… I currently live in an apartment, so I do not have that many activities”*. Furthermore, how the patients perceive the question may be complex, depending on the words used. Some emphasised that the word ‘activities’ is a term that can be perceived broadly and, therefore, is open to varying interpretations. The statement made from P12 indicates that every patient responds based on their perception of what the word ‘activities’ means to them, *“I respond based on what I do… spend time taking care of my horse, which takes up a lot of my day… Those who invented the question, what do they want to know? Is it about daily activities, grooming, or being able to clean the house and cook dinner?”*. In addition, a few patients stated that the words ‘pain’ and ‘functional impairment’ could be unintentionally misleading because it is automatically assumed that one or both are present, which is not necessarily true for everyone. P18 explained, *"You could have asked… when you think about all the activities you carry out in daily life, do you consider your current state satisfactory? I am already being led to believe that I should have pain and functional impairment”*. In continuation, some patients emphasised that the question contains several things they need to consider, which require additional attention. P15 said, *“There are actually many questions… taking into account all the activities you have during your daily life, that is one sentence. And then your level of pain is suddenly another question”*, and P16 said, *“One may have to read it twice or be very careful while reading it… There are many questions within the same question… One might mix it all a bit too much…. It might be a bit difficult to stay focused on what you are actually being asked about”*.

In summary, patients found the PASS anchor question important and relevant. They expressed that it may be complex to evaluate because they need to consider their daily life activities, pain level, and functional impairment in combination. However, when patients filled out the questionnaire and explained their reflections and how they make their choices, they seem to focus on daily activities in interaction with pain, which suggests that things may be difficult to explore separately. Finally, the lack of knee/hip-related clarification may lead some to respond based on their overall condition rather than the outcome of the knee/hip surgery explicitly.

PASS response options relevancy
Patients had different opinions when asked to consider the PASS response options, “1. Yes or 2. No”. A slight predominance of patients thought that the response options were precise and made sense when also considering the form of the question. P7 said, *“Yes and no, nothing can be placed in between, at least not from my perspective”*. In addition, none of the 18 patients had difficulty choosing a response option. However, some stated that other people could get confused if there were several options. P9 said, *“It can hardly be anything other than yes and no… it will be too difficult to add the response option ‘perhaps’… Because I think it will cause people to get more confused”.* However, almost as many patients suggested adding at least one more response option when considering other people than themselves. P18 said, *“I could imagine there would be others in between, in some grey area… if you want, you could write a comment. Or you could write ‘moderately’, or ‘I do not know’… something between yes and no”*. In summary, nearly half of the patients requested at least one additional response option for the PASS anchor question, but they did so by considering other people’s needs than themselves. Additionally, none of the 18 patients had difficulties choosing between the current response options.

**TF – Ambiguous wording**The following section describes how patients read and understood the TF anchor question and whether they found it relevant and meaningful. First, patients conceptualised the word ‘treatment’ differently. Second, some were concerned that the specific wording could be misunderstood. Finally, some thought the follow-up assessment timing predetermined their response choice. Furthermore, we must emphasise that patients would normally be asked to respond to the TF anchor question only if they responded ‘no’ to the PASS anchor question. However, in this study, we asked all 18 patients how they read and understood the TF anchor question independent of their response to the PASS anchor question.

TF anchor question relevancy and comprehensibility
Patients emphasised different things when responding to the TF anchor question, “Would you consider your current state as being so unsatisfactory that you think the treatment has failed?”. Approximately half of the patients considered their experienced knee/hip surgery outcome. They related the way they felt about their present condition when deciding whether they considered surgery to have failed. P14 described that the surgery had done nothing good, *“I must admit that I actually felt better before I had surgery… It is not to criticise anyone, that they have done anything wrong, but it has not done me any good”*. However, some patients related the question to more than their surgical outcome. The word ‘treatment’ was conceptualised in different ways, from the surgical intervention specifically to the whole treatment continuum. P11 said, *“It is a thumbs up, from surgeon to doctor, nurse, physiotherapists… I really feel like I have been treated properly”*. Additionally, P12 substantiated treatment as a combination of health care services, *“How I was received at the hospital department… Prelude to the surgery. The conversation with the doctor. The day of the surgery… The follow-up afterwards… the progress with the physiotherapist”*. Furthermore, some patients mentioned that the question concerned their expectations, whether they were fulfilled, and whether the treatment had been pointless. For example, P3 said, *“It is about if you do not think it turned out as originally intended”*, and P10 said, *“I think it is easy to understand. Whether it has been so unsatisfactory that you think it has been pointless”*.

Generally, patients had different opinions regarding the relevance and meaningfulness of the TF anchor question. Some thought it was a fair question to ask, which P18 talked about, *“It is a fair question to ask because some probably have not been as lucky as me. Who experiences that something is wrong with the prosthesis, where one leg is a bit longer, or still feel much pain”*. However, some of the patients undergoing knee/hip arthroplasty within the past 3 months thought the question was asked too early and would appreciate more time before responding, which P4 talked about, *“I have been prepared for the time it takes… Therefore, I cannot judge whether the treatment is satisfactory or has failed. I cannot decide anything yet”*. Additionally, some patients addressed that the question’s wording may create misunderstandings, leading to incorrect responses. Several patients explained a need to reread the question more than once to comprehend it fully. P16 talked about how the question could be misinterpreted because of its negative wording, *“And then I should not pick the response option ‘yes’. This was why I reread it again… It is a reverse question… therefore, you could make a mistake here too”*. Furthermore, some patients emphasised that they preferred the question to have positive rather than negative wording. P17 talked about how receiving a new knee or hip should be a positive experience, *“Somehow, I think it should be a positive experience (talks about the wording of the question). This is negative right away… I would rather have had the question, has it been a good experience? Are you happy with your experience?”*.

In summary, although patients confirmed that exploring why treatment fails is relevant, they interpret the TF anchor question differently. The focal point for their perception lies within the word ‘treatment’, which may vary from the surgical intervention specifically to the whole treatment continuum. In addition, some may have misinterpreted the question because it is negatively worded compared to the other questions. Finally, patients undergoing knee/hip arthroplasty within the past 3 months may have difficulties responding to the question because they consider it too early to judge if they think the treatment has failed. Consequently, patients questioned the relevancy of asking about failure within the first months following surgery because they were often not able to judge the outcome so soon after surgery, as they expected a recovery time of up to a year.

TF response options relevancy
In accordance with the PASS response options, patients had different opinions when considering the TF response options, “1. Yes or 2. No”. Approximately half thought that the options were precise and made sense when considering the form of the question. P10 said, *“It is not necessary to have something in between… Because the question asks directly if it has been unsatisfactory. Therefore, it is easy to respond whether it is a yes or a no”*. However, the other half of the patients suggested at least one more response option, which P11 talked about, *“I think that there could easily have been a middle response option. Because there will be many who will say that there are things they can do, and then there are things still missing”*.

Several patients undergoing knee/hip arthroplasty within the past 3 months discussed that their choice of response may be predetermined by the time of the follow-up assessment. P4 said that more time would have to pass before the question could be correctly responded to, *“If you wait 4, 5, 6 months after the surgery. I think you could get a correct response… This thing… I have gone through (talks about the surgery), was it worth the effort… I cannot say anything about that yet. And that is why I say ‘no’ to the question”*. In addition, some patients thought that to choose the response option ‘yes’, you must be in a condition equivalent to or worse than the condition preoperatively. For example, P12 explained that persistent pain and functional limitations would reflect treatment failure, *“Then you would still have problems with your knee. You would still not be able to walk much. And it would still be painful. You would actually be back to square one (talks about what it takes to respond ‘yes’ to the TF anchor question)”*. The single informant who responded ‘yes’ to treatment failure confirmed that the reason was precisely recurrent pain and limited functional improvement after surgery.

In summary, half of the patients felt the need for an additional TF response option, but their concern was based on considering others’ needs rather than their own. Similarly to what was found for the PASS anchor question, all 18 patients were able to choose a response between the current TF response options.

**Transversal analyses**The following four themes were generated inductively during the coding phase of working with the interview data. They emerged from the patient's perception of the three anchor questions and how they generally responded to the questionnaire.

**Temporal aspect**The temporal aspect found in the construct ‘treatment failure’ was not confirmed in questions asking about important change (MIC) or whether symptom levels were satisfactory (PASS). Since we identified a temporal aspect in how patients perceived and assessed the question about treatment failure, we wanted to investigate the aspect of ‘time’ in more detail to see if it, in general, affected the way patients responded.

This in-depth analysis confirmed that the postoperative time-point may have impacted the patients’ responses to the MIC and PASS anchor questions, but the way they read and understood the questions was constant. All patients undergoing knee/hip arthroplasty at our hospital receive follow-up assessments at 3, 12, and 24 months postoperatively. Most patients undergoing surgery within the past 3 months explained that their responses were likely to change during the upcoming months. P2 talked about how the condition would change between now and within 12 months, *“It depends a lot on the timing of when you receive this questionnaire… there is a big difference between the condition now and in, say, 12 months”*. This statement is expected from most recovering from surgery because of their developing condition. In addition, several patients indicated that they were informed by one or more clinicians before surgery that full recovery may take as long as 12 months, which helped reconcile their expectations. P4 talked about how long-term symptom improvement was expected despite the knee problems currently experienced, *“But I expect it to be temporarily (laughs). It should very much be so… I have been told it can take up to a year with a knee like this before I am back again”*.

In contrast to the TF anchor question, we did not find that patients altered their understanding and perception of the MIC and PASS anchor questions as time passed. It became evident that patients, independent of follow-up time point assessment, understood and perceived the MIC and PASS anchor questions similarly. However, most patients responding to the TF anchor question at 3 months questioned the relevance because they expected up to 12 months of recovery, which we previously described in the “ambiguous wording” theme. Patients responding at 3 months about whether they considered ‘treatment failure’ may have chosen the option ‘no’ simply because they were indecisive. This suggests that while the TF anchor question may not be appropriate for patients to respond to 3 months after knee/hip arthroplasty, the relevancy and understanding of the MIC and PASS anchor questions are not time-dependent.

Patients who recognise the questions may also have a similar understanding and perception as patients for whom the questions were unknown. The 18 informants were informed that if they received a routinely distributed follow-up questionnaire containing the three anchor questions currently under study, they should not respond until after our scheduled interview to avoid interference with their immediate perception of the questions. However, during the interview, three patients indicated that they had seen the questions before. For example, P3 said, *“I can see that it is the same questionnaire that was on the screen (…) It has been nice to go through it together, you and me”*, and P10 said, *“It must be what I have already filled out (talking about the questionnaire) that I should not have (laughs)”*. Since these patients made no comments about their understanding or perception of the questions changing during the interviews, it may suggest that they, in fact, perceive them similarly to the rest.

In summary, patients read and perceived the content of the MIC and PASS anchor questions constantly over time. Consequently, their responses to these questions about important change and satisfactory symptom states changed in correspondence with their perceived recovery after hip or knee arthroplasty. However, patients were indecisive about treatment failure and did not consider the question relevant at 3 months postoperatively because they found it too early to judge whether treatment had failed. Finally, a few patients had become unintentionally familiar with the questionnaire, but none desired to respond differently to any of the questions.

**Order and interrelations**We found that the order and interrelation of questions may help patients read and understand questions better and influence their perceptions of questions in general.

It became evident that questions and response options may be read and perceived in relation to each other. Patients are, consciously and unconsciously, likely to compare questions and response options to gain perspective on things that create meaning for them and things they may not comprehend. During the interviews, several patients explicitly compared the PASS and TF anchor questions. For example, P1 explained, *“Number 15 is designed better than number 14 (said about the TF anchor question compared to the PASS anchor question)”*, and P16 said, *“It is easier to comprehend than question 14. Because it is only one question… If there had also been something about subsequent rehabilitation, you would add more questions to it (said about the TF anchor question compared to the PASS anchor question)”*. Thus, some patients thought it was easier to comprehend a question when designed to ask about one specific thing. In addition, it should be considered that varying complexity in understanding questions may lead to frustration among some patients trying to respond. For example, P2 compared two questions with varying complexity, *“How can it be so simple (pointing at the PASS anchor question) when this (pointing to the MIC anchor question) is so… honestly… It does not make sense to me”*. This statement suggests that some patients may find it challenging to understand why questionnaires include questions that vary in complexity.

The order in which patients are introduced to the questions can be as crucial as the interrelation between questions. P5 talked about how the MIC anchor question, in many ways, concerns the same things as the Oxford Hip Score questionnaire, *“And that is, in fact, similar to what I have responded in the first questions (refer to the 12 Oxford Hip Score questions)”*. Furthermore, several patients emphasised that a few of the 15 questions stood out and were designed differently. P4 talked about how the PASS and TF response options were different compared to the options from the previous questions, *“It is an entirely different type of response options (talking about the PASS and TF anchor questions) … it could probably have been five options as well… that might add some gradation to it”*. These examples may indicate that patients form an expectation of the following questions in a questionnaire or survey that is based on already completed questions.

In summary, patients’ perceptions of questions and ways of responding to them may occur by comparing them to each other, and some may be negatively affected by varying complexity. Furthermore, some patients suggest that it would be preferable to have familiarity and consistency. However, whether this could lead to responses becoming too predictable is debatable, causing individuals to respond automatically rather than address the specific question.

**Emotions**We found that patients’ perceptions and opinions about questions and response options may be impacted by the emotions they experience when responding. The emotions were related to the need to elaborate or because they did not feel the response options were spot on.

Some patients were looking for a chance to elaborate their responses because sometimes they felt that the response options were inadequate or imprecise, considering their current situation. Therefore, several patients explained that it should be possible to supplement questions with additional comments using a text field. P12 explained, *“Maybe if you could elaborate it a bit, like right now where we sit and talk together… that you would be able to write down a bit of text”*. In addition, some patients expressed why they were generally not fond of responding to questionnaires because they felt they were not allowed to explain their responses sufficiently. P14 said, *“This is why I have been so much against all these questionnaires because I do not feel they can describe specifically what I mean. You cannot always do that with yes and no questions, and how is it from 1 to 10 response options”*. In summary, emotions are related to responding to questionaries, but it is difficult to determine in what way these emotions affect the way patients respond. If they get annoyed, do they, e.g. respond more randomly and with less consideration? As a result, some propose including an optional text field to provide additional context and express their emotions when responding.

**Current health state**We identified that patients’ perceptions and opinions about questions may be impacted by their current health state, which shapes and influences their experience when responding.

Health-related issues may affect patients’ perception and their reason for responding to a question in a certain way. In our study, some patients emphasised that previous or subsequent surgeries in the opposite leg may have affected their responses to questions. P14 talked about a recent operation that made it difficult to distinguish one hip from the other, *“It can be difficult to distinguish between the right and left hip because now I have severe pain across my lower back. It is the left hip, in particular, causing the problem, and it is the most recent surgery I have undergone”*. Additionally, some patients experienced unforeseen complications such as infections or re-operations. For example, P10 talked about a complication after infection, *“I would say that the complication I have had… it has hampered me to a great extent. It has taken twice as long to heal. It is actually only within the last 14 days that I have felt any improvement”*. Furthermore, it must be highlighted that health state issues could be related to more than patients’ surgeries. Patients may consider it challenging to separate symptoms and treatment effects in one joint when also experiencing discomfort in the opposite or other joints. P7 said, *“I try only to consider the left knee, but sometimes it can be difficult to distinguish between them both”*.

Similarly, it was highlighted that age-related health issues could impact some of the patient responses to questions. According to P15, people sometimes reach an age where, for example, their balance is not as good as it used to be, which causes functional limitation considerations, *“I would say that what is happening now is age-related. My balance now is not what it used to be when using a ladder”*. In addition, a few of the oldest patients emphasised that the number and impact of health-related issues increase with age. P1 said, “*But I am also very old, 86. Something always comes around when you are this old (referring to illnesses and general health problems)”*. In summary, patient's current health state may influence their perceptions and responses to questions, meaning that they may have difficulties judging the outcome of their recent knee/hip arthroplasty without simultaneously mixing it with other health problems. In addition, patients may also find it difficult to separate their perceptions and responses to questions explicitly targeting their recent knee/hip arthroplasty if they suffer from other and perhaps more severe health-related issues.
