# Supplementary material for: Anchor questions to improve patient-reported outcome measure interpretability in patients undergoing knee or hip arthroplasty - a mixed-methods content validity, construct validity, and reliability study
Source: Qual Life Res. 2025 May 16;34(8):2279–91. doi: 10.1007/s11136-025-03987-y (PMC12274218; doi:10.1007/s11136-025-03987-y)
Supplement: Supplementary file 6 — Supplementary Material 6 [file 11136_2025_3987_MOESM6_ESM.docx]

**Online Resource 4**

**Article title**Anchor questions to improve patient-reported outcome measure interpretability in patients undergoing knee or hip arthroplasty – A mixed-methods content validity, construct validity, and reliability study

**Journal name**Quality of Life Research

**Author names**
Lasse K. Harris^1,2^, Trine S. Larsen^1,3,4^, Berend Terluin^5,6^, Henrik H. Lauridsen^7^, Anders Troelsen^1,2^,
Lina H. Ingelsrud^1^

**Affiliations**
^1^ Department of Orthopaedic Surgery, Copenhagen University Hospital Hvidovre, Copenhagen, Denmark
^2^ Department of Clinical Medicine, Faculty of Health and Medical Sciences, University of Copenhagen, Denmark
^3^ Department of Clinical Research, Copenhagen University Hospital, Hvidovre, Copenhagen, Denmark
^4^ Department of People and Technology, Roskilde University, Roskilde, Denmark
^5^ Department of General Practice, Amsterdam UMC Location, Vrije Universiteit Amsterdam, the Netherlands
^6^ Amsterdam Public Health Research Institute, Amsterdam, the Netherlands
^7^ Department of Sports and Clinical Biomechanics, University of Southern Denmark, Odense, Denmark

**Corresponding author**Lasse K. Harris, E-mail: [lasse.kindler.harris@regionh.dk](mailto:lasse.kindler.harris@regionh.dk)

| Knee arthroplasty  3 months: n = 1423 between April 2018 and September 2022  12 months: n = 2207 between February 2016 and December 2021  24 months: n = 2041 between February 2016 and December 2020 | | | | |  |  | Hip arthroplasty  3 months: n = 1197 between April 2018 and September 2022  12 months: n = 1301 between July 2017 and December 2021  24 months: n = 1458 between July 2016 and December 2020 | | | | | |  |  |
| --- | --- | --- | --- | --- | --- | --- | --- | --- | --- | --- | --- | --- | --- | --- |
|  | |  | | |  |  |  | |  | | | |  |  |
|  | |  | Excluded preoperative questionnaire missing 3 months: n = 262  12 months: n = 470  24 months: n = 442 | | | | |  |  | | Excluded preoperative questionnaire missing 3 months: n = 229 12 months: n = 279  24 months: n = 315 | | | |
|  |  |  |  |  |  |  |  |  |  | |  |  |  |  |
|  | |  | | |  |  |  | |  | | | |  |  |
| Patients with completed preoperative questionnaire  3 months: n = 1161  12 months: n = 1737  24 months: n = 1599 | | | | |  |  | Patients with completed preoperative questionnaire  3 months: n = 968 12 months: n = 1022  24 months: n = 1143 | | | | | |  |  |
|  | |  | | |  |  |  | |  | | | |  |  |
|  | |  | Excluded  Postoperative questionnaire missing 3 months: n = 113  12 months: n = 218  24 months: n = 251 | | | | |  |  | | Excluded  Postoperative questionnaire missing 3 months: n = 102 12 months: n = 136  24 months: n = 183 | | | |
|  |  |  |  |  |  |  |  |  |  | |  |  |  |  |
|  | |  | | |  |  |  | |  | | | |  |  |
| Patients with completed postoperative questionnaire  3 months: n = 1048  12 months: n = 1519  24 months: n = 1348 | | | | |  |  | Patients with completed postoperative questionnaire  3 months: n = 866 12 months: n = 886  24 months: n = 960 | | | | | |  |  |
|  |  | | | |  |  |  | | |  | | |  |  |
|  |  | | | Excluded - missing OKS or anchor  3 months: n = 8  12 months: n = 21  24 months: n = 11 | | | |  | |  | | Excluded - missing OHS or anchor  3 months: n = 9  12 months: n = 0  24 months: n = 5 | | |
|  |  | | |  |  |  |  |  | |  | |  |  |  |
|  |  | | | |  |  |  | | |  | | |  |  |
| Patients with complete data for primary analyses 3 months: n = 1040 (73%)  12 months: n = 1499 (68%)  24 months: n = 1337 (66%) | | | | |  |  | Patients with complete data for primary analyses 3 months: n = 857 (72%) 12 months: n = 886 (68%)  24 months: n = 955 (66%) | | | | | |  |  |
| **Online Resource Figure 1.** Flow chart of patients enrolled from the local registry. OKS; Oxford Knee Score; OHS, Oxford Hip Score. | | | | | | | | | | | | | | |
