# Supplementary material for: Anchor questions to improve patient-reported outcome measure interpretability in patients undergoing knee or hip arthroplasty - a mixed-methods content validity, construct validity, and reliability study
Source: Qual Life Res. 2025 May 16;34(8):2279–91. doi: 10.1007/s11136-025-03987-y (PMC12274218; doi:10.1007/s11136-025-03987-y)
Supplement: Supplementary file 7 — Supplementary Material 7 [file 11136_2025_3987_MOESM7_ESM.docx]

**Online Resource 5**

**Article title**Anchor questions to improve patient-reported outcome measure interpretability in patients undergoing knee or hip arthroplasty – A mixed-methods content validity, construct validity, and reliability study

**Journal name**Quality of Life Research

**Author names**
Lasse K. Harris^1,2^, Trine S. Larsen^1,3,4^, Berend Terluin^5,6^, Henrik H. Lauridsen^7^, Anders Troelsen^1,2^,
Lina H. Ingelsrud^1^

**Affiliations**
^1^ Department of Orthopaedic Surgery, Copenhagen University Hospital Hvidovre, Copenhagen, Denmark
^2^ Department of Clinical Medicine, Faculty of Health and Medical Sciences, University of Copenhagen, Denmark
^3^ Department of Clinical Research, Copenhagen University Hospital, Hvidovre, Copenhagen, Denmark
^4^ Department of People and Technology, Roskilde University, Roskilde, Denmark
^5^ Department of General Practice, Amsterdam UMC Location, Vrije Universiteit Amsterdam, the Netherlands
^6^ Amsterdam Public Health Research Institute, Amsterdam, the Netherlands
^7^ Department of Sports and Clinical Biomechanics, University of Southern Denmark, Odense, Denmark

**Corresponding author**Lasse K. Harris, E-mail: [lasse.kindler.harris@regionh.dk](mailto:lasse.kindler.harris@regionh.dk)

| **Supplementary table.** Patient preoperative characteristics for exploring construct validity and reliability for 3-, 12-, and 24-month knee surgery cohorts.  Numbers are median (2.5-97,5% quantile range) unless otherwise stated *. A responder analysis. | | | | | | | | | | |
| --- | --- | --- | --- | --- | --- | --- | --- | --- | --- | --- |
| **Factor** | | 3-month  responders n = 1040 | 3-month non-responders n = 383 | P-value | 12-month responders n = 1499 | 12-month non-responders n = 708 | P-value | 24-month responders n = 1337 | 24-month  non-responders n = 704 | P-value |
| Age | | 70 (62-75) | 71 (62-76) | 0.44 | 69 (61-75) | 69 (61-75) | 0.88 | 69 (62-74) | 69 (60-75) | 0.80 |
| Sex % (n) female | | 59% (614) | 59% (227) | 0.99 | 58% (875) | 63% (444) | 0.06 | 59% (782) | 61% (431) | 0.25 |
| BMI | | 29 (26-33) | 29 (26-34) | 0.49 | 29 (26-33) ^a^ | 29 (26-34) ^a^ | 0.01 | 29 (26-33) ^a^ | 29 (26-35) ^a^ | 0.01 |
| ASA classification % (n) | |  |  |  |  |  |  |  |  |  |
|  | 1 | 7.2% (75) | 7.1% (27) | 0.04 | 15.1% (129) | 8.6% (61) | 0.56 | 15.3% (146) | 7.7% (54) | 0.06 |
|  | 2 | 73.4% (763) | 67.1% (257) |  | 63.2% (542) | 71.2% (504) |  | 67.4% (644) | 71.9% (506) |  |
|  | 3 | 19.1% (199) | 25.8% (99) |  | 21.6% (185) | 20.1% (142) |  | 17.1% (163) | 20.3% (143) |  |
|  | 4 | 0.3% (3) | - |  | 0.1% (1) | 0.1% (1) |  | 0.2% (2) | 0.1% (1) |  |
| OKS | | 22 (17-27) | 20 (14-25) ^c^ | 0.01 | 22 (18-27) | 18 (14-23) ^g^ | 0.01 | 22 (18-27) | 19 (14-24) ^j^ | 0.01 |
| EQ-5D-3L | | 0.66 (0.59-0.72) ^a^ | 0.66 (0.39-0.72) ^d^ | 0.04 | 0.70 (0.59-0.72) ^b^ | 0.63 (0.33-0.72) ^g^ | 0.01 | 0.66 (0.59-0.72) ^b^ | 0.66 (0.33-0.72) ^j^ | 0.01 |
| EQ VAS | | 66 (50-80) ^b^ | 60 (43-79) ^e^ | 0.01 | 70 (50-80) ^f^ | 60 (41-73) ^h^ | 0.01 | 70 (50-80) ^i^ | 51 (41-72) ^j^ | 0.01 |
| ^*^ Wilcoxon Signed Rank test for continues variables and chi-square test for dichotomous variables. ^a^ Missing data n = 1, ^b^ Missing data n = 2, ^c^ Missing data n = 243, ^d^ Missing data n = 244,  ^e^ Missing data n = 247, ^f^ Missing data n = 6, ^g^ Missing data n = 451,  ^h^ Missing data n = 452,  ^i^ Missing data n = 7 , ^j^ Missing data n = 423. BMI; Body Mass Index, ASA; American Society of Anaesthesiologists physical status classification system, 1; normal health, 2; mild systemic disease, 3; severe systemic disease, 4; severe systemic disease that is a constant threat to life, OKS; Oxford Knee Score, VAS; Visual Analog Scale. | | | | | | | | | | |

| **Supplementary table.** Patient preoperative characteristics for exploring construct validity and reliability for 3-, 12-, and 24-month hip surgery cohorts.  Numbers are median (2.5-97,5% quantile range) unless otherwise stated *. | | | | | | | | | | |
| --- | --- | --- | --- | --- | --- | --- | --- | --- | --- | --- |
| **Factor** | | 3-month responders n = 857 | 3-month non-responders n = 340 | P-value | 12-month responders n = 886 | 12-month non-responders n = 415 | P-value | 24-month responders n = 955 | 24-month non-responders n = 503 | P-value |
| Age | | 70 (62-76) | 72 (61-79) | 0.05 | 70 (62-76) | 71 (61-78) | 0.15 | 70 (61-75) | 71 (62-78) | 0.03 |
| Sex % (n) female | | 57% (490) | 64% (217) | 0.04 | 56% (497) | 65% (269) | 0.01 | 58% (550) | 62% (312) | 0.11 |
| BMI | | 27 (24-31) | 27 (25-32) | 0.37 | 27 (24-31) | 27 (25-31) | 0.77 | 27 (24-30) | 27 (24-31) | 0.74 |
| ASA classification % (n) | |  |  |  |  |  |  |  |  |  |
|  | 1 | 15.1% (129) | 8.2% (28) | 0.01 | 15.1% (134) | 10.3% (43) | 0.01 | 15.3% (146) | 11.7% (59) | 0.04 |
|  | 2 | 63.2% (542) | 64.1% (218) |  | 65.8% (583) | 62.2% (258) |  | 67.4% (644) | 65.8% (331) |  |
|  | 3 | 21.6% (185) | 26.5% (90) |  | 19.1% (169) | 27.0% (112) |  | 17.1% (163) | 22.3% (112) |  |
|  | 4 | 0.1% (1) | 1.2% (4) |  | - | 0.5% (2) |  | 0.2% (2) | 0.2% (1) |  |
| OHS | | 21 (16-26) | 17 (12-24) ^b^ | 0.01 | 21 (16-27) | 18 (12-25) ^c^ | 0.01 | 22 (17-27) | 18 (12-26) ^d^ | 0.01 |
| EQ-5D-3L | | 0.66 (0.56-0.72) | 0.63 (0.33-0.66) ^b^ | 0.01 | 0.66 (0.56-0.72) | 0.59 (0.32-0.66) ^c^ | 0.01 | 0.66 (0.56-0.72) | 0.66 (0.32-0.72) ^d^ | 0.01 |
| EQ VAS | | 60 (45-78) ^a^ | 50 (34-68) ^b^ | 0.01 | 60 (49-80) ^a^ | 50 (35-72) ^c^ | 0.01 | 61 (50-80) ^a^ | 50 (40-75) ^e^ | 0.01 |
| ^*^ Wilcoxon Signed Rank test for continues variables and chi-square test for dichotomous variables. ^a^ Missing data n = 2, ^b^ Missing data n = 217, ^c^ Missing data n = 279, ^d^ Missing data n = 315,  ^e^ Missing data n = 316. BMI; Body Mass Index, ASA; American Society of Anaesthesiologists physical status classification system, 1; normal health, 2; mild systemic disease, 3; severe systemic disease, 4; severe systemic disease that is a constant threat to life, OHS; Oxford Hip Score, VAS; Visual Analog Scale. | | | | | | | | | | |
