# Supplementary material for: Anchor questions to improve patient-reported outcome measure interpretability in patients undergoing knee or hip arthroplasty - a mixed-methods content validity, construct validity, and reliability study
Source: Qual Life Res. 2025 May 16;34(8):2279–91. doi: 10.1007/s11136-025-03987-y (PMC12274218; doi:10.1007/s11136-025-03987-y)
Supplement: Supplementary file 8 — Supplementary Material 8 [file 11136_2025_3987_MOESM8_ESM.docx]

**Online Resource 6**

**Article title**Anchor questions to improve patient-reported outcome measure interpretability in patients undergoing knee or hip arthroplasty – A mixed-methods content validity, construct validity, and reliability study

**Journal name**Quality of Life Research

**Author names**
Lasse K. Harris^1,2^, Trine S. Larsen^1,3,4^, Berend Terluin^5,6^, Henrik H. Lauridsen^7^, Anders Troelsen^1,2^,
Lina H. Ingelsrud^1^

**Affiliations**
^1^ Department of Orthopaedic Surgery, Copenhagen University Hospital Hvidovre, Copenhagen, Denmark
^2^ Department of Clinical Medicine, Faculty of Health and Medical Sciences, University of Copenhagen, Denmark
^3^ Department of Clinical Research, Copenhagen University Hospital, Hvidovre, Copenhagen, Denmark
^4^ Department of People and Technology, Roskilde University, Roskilde, Denmark
^5^ Department of General Practice, Amsterdam UMC Location, Vrije Universiteit Amsterdam, the Netherlands
^6^ Amsterdam Public Health Research Institute, Amsterdam, the Netherlands
^7^ Department of Sports and Clinical Biomechanics, University of Southern Denmark, Odense, Denmark

**Corresponding author**Lasse K. Harris, E-mail: [lasse.kindler.harris@regionh.dk](mailto:lasse.kindler.harris@regionh.dk)

| **Supplementary table.** Present state bias (PSB) and model fit indices, and reliability for minimal important change (MIC) anchor question and the Oxford Knee and Hip Score (Change) Scores at 3, 12, and 24 months after knee or hip arthroplasty. A sensitivity analysis of measurement variance. | | | | | | |
| --- | --- | --- | --- | --- | --- | --- |
| **Factor** | **Knee arthroplasty** | | | **Hip arthroplasty** | | |
|  | 3 months n = 1039 | 12 months n = 1490 | 24 months n = 1333 | 3 months n = 857 | 12 months n = 886 | 24 months n = 952 |
| **MIC** |  |  |  |  |  |  |
| PSB ^a^ | 0.57 (0.44 - 0.70) ^b^ | 0.73 (0.59 - 0.87) ^b^ | 0.58 (0.44 - 0.73) ^b^ | 0.52 (0.32 - 0.72) ^c^ | 0.55 (0.29 - 0.81) ^c^ | 0.53 (0.28 - 0.79) ^c^ |
| CFI | 0.961 | 0.979 | 0.979 | 0.949 | 0.964 | 0.974 |
| TLI | 0.957 | 0.977 | 0.978 | 0.947 | 0.962 | 0.972 |
| RMSEA | 0.057 | 0.047 | 0.048 | 0.066 | 0.061 | 0.054 |
| ^a^ Values in parentheses are 95% confidence interval, calculated using 1000-replication bootstrapping and reported as 0.025-to-0.975 quantiles.  ^b^ Factor loading constrained items 2, 4, 5, 8, 9, 10, 11, 12, and threshold constrained items 5, 8, 9, 10, 12.  ^c^ Factor loading constrained items 2, 3, 4, 5, 6, 8, 10, 11, 12, and threshold constrained items 2, 3, 4, 6, 8, 10, 11, 12. CFI; comparative fit index, TLI; Tucker-Lewis index, RMSEA; root mean square error of approximation. | | | | | | |
